# A Time Series Approach to Random Number Generation: Using Recurrence Quantification Analysis to Capture Executive Behavior

**DOI:** 10.3389/fnhum.2015.00319

**Published:** 2015-06-05

**Authors:** Wouter Oomens, Joseph H. R. Maes, Fred Hasselman, Jos I. M. Egger

**Affiliations:** ^1^Centre of Excellence for Neuropsychiatry, Vincent van Gogh Institute for Psychiatry, Venray, Netherlands; ^2^Donders Institute for Brain, Cognition and Behavior, Radboud University, Nijmegen, Netherlands; ^3^Learning and Plasticity, Behavioral Science Institute, Radboud University, Nijmegen, Netherlands; ^4^School of Pedagogical and Educational Science, Radboud University, Nijmegen, Netherlands

**Keywords:** random number generation, recurrence quantification analysis, executive functioning, cognition, principal component analysis

## Abstract

The concept of executive functions plays a prominent role in contemporary experimental and clinical studies on cognition. One paradigm used in this framework is the random number generation (RNG) task, the execution of which demands aspects of executive functioning, specifically inhibition and working memory. Data from the RNG task are best seen as a series of successive events. However, traditional RNG measures that are used to quantify executive functioning are mostly summary statistics referring to deviations from mathematical randomness. In the current study, we explore the utility of recurrence quantification analysis (RQA), a non-linear method that keeps the entire sequence intact, as a better way to describe executive functioning compared to traditional measures. To this aim, 242 first- and second-year students completed a non-paced RNG task. Principal component analysis of their data showed that traditional and RQA measures convey more or less the same information. However, RQA measures do so more parsimoniously and have a better interpretation.

## Introduction

In experimental and clinical studies on cognition, the concept of “executive functions” plays a dominant role [see e.g., Jurado and Rosselli (2007), for an overview]. Executive functioning is generally used as an umbrella term to refer to a set of higher-order cognitive processes that allow an individual to exert control over lower cognitive processes. However, discussions about the exact nature of these higher-order processes and their neural correlates are ongoing (e.g., Gilbert and Burgess, 2008; McCabe et al., 2010; Packwood et al., 2011; Tsuchida and Fellows, 2013; Anastas et al., 2014).

One task that has seen some use in the investigation of executive functioning is the random number generation (RNG) task. In this task, participants are instructed to generate a random sequence of letters or digits. Executive functioning can be assessed using this task by measuring departures from randomness. Specifically, the observed order in human-generated sequences is often attributed to imperfections of the central executive and working memory (Baddeley, 1966; Brugger, 1997; Baddeley et al., 1998), in particular to the inability to inhibit stereotyped (i.e., repetitive) behavior and to monitor and update recent responses (Towse and Neil, 1998; Miyake et al., 2000; Friedman and Miyake, 2004). This inability to be random is operationalized using a series of randomization measures that capture different types of order (Evans, 1978; Towse and Neil, 1998). These measures typically align into three factors: inhibition of prepotent responses, working memory updating, and output inhibition (Towse and Neil, 1998; Peters et al., 2007; Maes et al., 2011). Both inhibition and updating refer to sequential processes and, therefore, these factor labels imply that the selection of the next number in a human-generated sequence is inherently contextual, i.e., it is a function of previously selected numbers (Brugger, 1997; Schulz et al., 2012). For example, neither inhibition nor updating can exist in a vacuum of only a single “behavior” (in the case of RNG, a single digit). A participant can only inhibit a digit, or a sequence of digits, when the same digit or sequence has been used before. Accordingly, the entire sequence can be seen as a time series of human executive behavior.

However, contrary to the assumption of a determinate change process being at least partly responsible for generating the trial series of “random” numbers, most of these randomization measures calculated from the series are state-based summary statistics referring to the deviation of independent event occurrence from stochastic randomness. Therefore, all the information about potential non-random state-propagation rules involved in number selection is lost (Gilden, 2001). For example, an often used measure of randomness is RNG (Evans, 1978; Towse and Neil, 1998). RNG describes the difference between the observed distribution of digrams, regardless of the order in which these digrams occur, compared to an uniform distribution of digrams. The same method applies to most other randomization measures. Schulz et al. (2012) demonstrated the importance of time-evolutionary information in RNG by predicting one participant’s number selection based on the sequence of another participant, and these predictions became more precise when longer sequences were used. This corresponds with growing evidence that variability in behavioral data is not mere random fluctuation (Gilden, 2001; Van Orden et al., 2003) but an essential constituent of the understanding of human (executive) behavior (Riley and Turvey, 2002). Hence, RNG may be best analyzed by non-linear methods that require no assumptions about the nature of the data in question, but quantify the characteristics of any temporal pattern in the sequence of numbers (Shockley, 2005; Webber and Zbilut, 2005).

**Figure 1:**
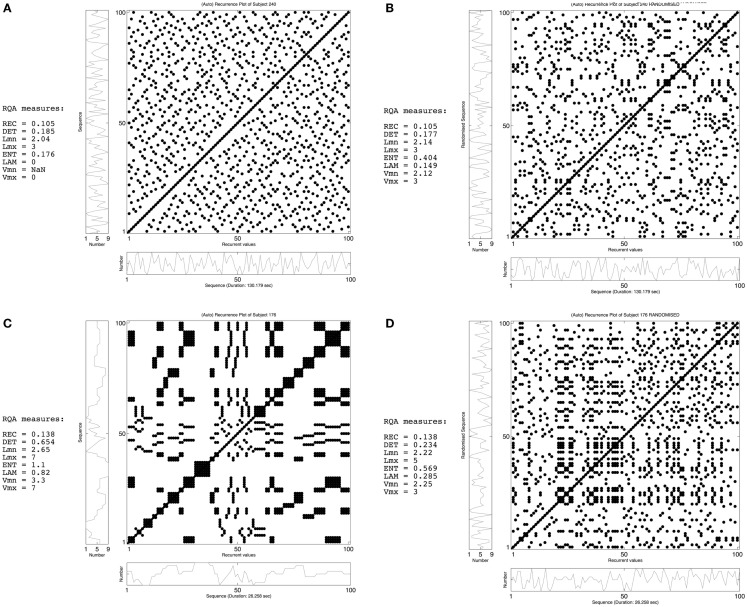
**Recurrence plots (RPs) of the RNG task of participant 240 (upper row) and participant 176 (bottom row)**. The left-side panels (**A,C**) display the observed time-series of both participants, while the right-side panels (**B,D**) show a randomized version of the same sequence. The RQA measures demonstrate the effect of randomizing the temporal order of the time-series: for both participants the recurrence rate is the same, but the formation of line structures differs from the observed sequence to the randomized version (see main text for a more detailed explanation on the interpretation of RPs and RQA).

Recurrence plots (RPs) and recurrence quantification analysis (RQA) are such non-linear approaches to time series analysis that exploit the dynamical organization of the entire time series (Webber and Zbilut, 1996, 2005). RPs display easily interpretable information about pattern recurrences on different time scales (Eckman et al., 1987), while RQA is an objective quantification of those recurrent patterns that can be associated with properties of non-linear dynamical systems (Webber and Zbilut, 1994; Marwan et al., 2007). In the current study, we use auto-RPs, which are a visual representation of a time series compared to itself. This visual representation is created by plotting the time series *x* on both axes in an *N* ×* N* square matrix, where *N* is the length of the time series and *x*(*i*) is the *i*th measure in the time series. In case of random number sequences, a dot is placed at (*i, j*), whenever *x*(*j*) has the same number as *x*(*i*). Hence, dots are placed at the same moment in time, alongside the diagonal *i* = *j*, and at every moment later in time when the same number recurs. Due to the auto-recurrent nature, these RPs are symmetrical regarding the diagonal *i* = *j* and both planes contain the same information (see Figure 1). Using RQA, this information is described by the following variables: recurrence rate, determinism, longest diagonal line length, entropy, laminarity, and trapping time. Recurrence rate quantifies the amount of recurrent points or dots (excluding the diagonal), expressed as a percentage. In other words, recurrence rate is the proportion dots to non-dots in the RP. These recurrent points can be either scattered across the RP or clustered in diagonal and vertical line structures. Determinism measures the proportion of recurrent points forming diagonal line structures and quantifies the amount of repetitive patterns. Shorter diagonal line structures equal unstable patterns (more chaotic patterns) indicated by the longest diagonal line length. Shannon information entropy is based on a histogram of all diagonal line lengths present in the data and is an index of the complexity of the deterministic structure of the time series (Pellecchia and Shockley, 2005; Webber and Zbilut, 2005). Finally, laminarity measures the proportion of recurrent points forming vertical line structures, whereas trapping time is the average length of vertical line structures (Marwan et al., 2002). These vertical line structures are indicative of patterns that are trapped in one state (repeating the same behavior over and over), while trapping time quantifies the stability of these trapped states.

Figure 1 shows two examples of how temporal sequences are quantified using RPs and RQA. The left-side panels display the observed time series of two participants, while the right-side panels show shuffled versions of both time series. The upper left panel (Figure 1A) displays a time series with dots scattered across the entire surface of the RP and only a few of these dots form diagonal and vertical line structures. This minimal amount of line structures equals the absence of (complex) repetitive behavior, which is reflected by a low value for determinism and laminarity and a short diagonal line length. On the other hand, the lower left RP (Figure 1C) displays a highly clustered and, therefore deterministic time series, reflected in both the formation of diagonal and vertical line structures and a high value for determinism and laminarity. The lower right RP (Figure 1D) is a randomly shuffled version of the same time series. By shuffling the time series, most line structures disappear, which is quantified by a drop in determinism and laminarity. Furthermore, this drop in determinism is indicative of the disappearance of long-range correlations and, therefore, the importance of keeping time series intact. Although this does not absolutely refute the use of conventional measures, it does point toward the use of non-linear methods and, hence, RQA as the better method.

Combined, these RQA measures expose the dynamical processes inherent to the construct under study. RQA has been applied successfully in the field of postural control (Riley et al., 1998; Pellecchia and Shockley, 2005), eye movement coordination (Richardson et al., 2007; Dale et al., 2010), motor control (Wijnants et al., 2009), problem solving cognition (Stephen et al., 2009), and treatment efficacy for aggressive children (Lichtwarck-Aschoff et al., 2012).

The goal of the current study was to explore the utility of RQA in quantifying number sequences generated in the framework of RNG tasks. In a typical RNG procedure, responses of participants are paced using a metronome. However, recent research demonstrated a coupling of behavior between the metronome and several human systems, resulting in a change of long-range correlations within time series (Coey et al., 2014; Marmelat et al., 2014; Rigoli et al., 2014). For the current study, this coupling would imply that both motor behavior and number selection is influenced by the variability and speed of the metronome. Consistently, a positive correlation between metronome interval length and the quality of randomness is often reported (Baddeley, 1966; Brugger, 1997; Joppich et al., 2004). Withal, state-based randomization measures should not be as much affected by a change in long-range correlations. In contrast, RQA measures are sensitive to the sequential order of time series, and to eliminate this coupling effect, we used a non-paced RNG paradigm to examine the utility of RQA over traditional measures. Since little to no research has been done on RNG without a pacing signal, we first explored the similarities of the current results with those from a paced version. Specifically, we compared the factorial structure of the current data with results reported in the literature (Maes et al., 2011). Next, we added RQA measures to our factorial structure, to determine their similarity to the original RNG randomization measures and their interpretability within the often-used theoretical threefold of executive functioning: shifting between mental sets, updating and monitoring of working memory contents, and inhibition of prepotent responses (Miyake et al., 2000). Finally, we explored whether RQA provides a better explanation of executive functioning as measured by the RNG paradigm than is the case for the more conventional randomness measures. This was achieved by comparing the RQA measures to the randomization measures in terms of their interpretability, the number of components in the model, and the amount of variance explained.

## Materials and Methods

### Participants

A group of 242 first- and second-year students attending courses in methodology and statistics completed the RNG task. These students were asked during their respective classes to participate without getting any compensation. The task was completed within the classroom environment.

### Apparatus

Participants generated a sequence of numbers by clicking (with a standard input device) on the cells of a 3 × 3 (12.5 × 13 cm) grid, shown on a laptop computer. The digits were displayed in a vertically inversed num-pad grid, with the numbers 1, 2, and 3 in the top row. Each response resulted in the appearance of a 3.8 × 4.2-cm rectangle drawn with a dotted line in the corresponding cell of the grid, while the grid and digits remained visible throughout. This rectangle disappeared upon selection of the subsequent response, and the newly selected digit was again marked by the appearance of a dotted-line rectangle. The grid was drawn in black against a white background.

### Procedure

Participants were orally given the instruction to generate 100 numbers in their own pace with the sole rule that the numbers must be as random as possible. Since all participants were taught in basic methodology and statistics, no further explanations on the meaning of random was given. Because participants were free to take the time they needed to produce the number sequence, we term this procedure non-paced *RNG*. In contrast, a paced *RNG* paradigm uses a fixed decision time interval (e.g., one response each second) to randomly select numbers.

### Measures

The randomization measures used in the current study were identical to those originally described by Towse and Neil (1998) and were calculated using software that these authors developed: RgCalc. The measures used were: adjacency, coupon, Phi 2-gram, Phi 3-gram, Phi 4-gram, Phi 5-gram, Phi 6-gram, Phi 7-gram, redundancy, repetition gap (mean), repetition gap (median), repetition gap (mode), RNG, RNG 2, runs, and Turning Point Index (TPI). For a full explanation of these measures, see Towse and Neil (1998) and Maes et al. (2011).

The RQA measures were calculated using Cross Recurrence Plot toolbox for MATLAB (Marwan et al., 2007). The measures were recurrence rate, determinism, longest diagonal line length, averaged diagonal line length entropy, laminarity, and trapping time (see Introduction for a full explanation of these measures). In many cases, laminarity proved to be zero, which makes it impossible to calculate trapping time for these cases. In order to include all participants in our principal component analysis (PCA), the missing trapping times were set to zero. These RQA measures are affected by the chosen embedding dimension (*M*), time delay (τ), minimal line length, and radius. To optimize the information gained from the time series, *M* is chosen based on the disappearance of false nearest neighbors (Kennel et al., 1992) and τ on the first minimum in the mutual information (Fraser and Swinney, 1986). However, since random number sequences contain only nominal information, we used the same embedding dimension (*M* = 1) as practiced by Coco and Dale (2014), Dale et al. (2010), and Dale and Spivey (2005) on categorical time series data. Our parameter τ was set to 1, which is advised for discontinuous data (Webber and Zbilut, 2005). Finally, the minimal line length was set to 2, to ensure that every recurring combination of 2 or more digits are considered a diagonal or vertical line structure, and the radius was set to less than 1 such that only exact matches are considered recurrent (Orsucci et al., 1999).

### Analysis

To compare the current data to earlier results, the same data-reduction analysis (IBM SPSS Statistics 19.0) was used as in the study by Maes et al. (2011): PCA with uncorrelated components (varimax rotation) based on eigenvalues >1. Next, to determine the similarity of the randomization and RQA measures, factor analysis was performed on the combined data from the current experiment and those from Maes et al. (Experiment 1). However, because there is no reason to assume that inhibition of prepotent responses, updating, and output inhibition are not inter-correlated, four promax rotated components were sampled. Finally, the factorial structure based on the RQA measures was compared to that based on the randomization measures, focusing on both the number of components and the proportion of explained variance. To be able to compare the proportion of variance explained, the analysis was kept identical to the aforementioned dimension reduction technique: PCA with uncorrelated components (varimax rotation) based on eigenvalues >1.

## Results

To examine the factorial similarity of the current data and earlier results, PCA was conducted on all 16 aforementioned randomization measures with orthogonal rotation (varimax). The Kaiser–Meyer–Olkin measure verified the sampling adequacy for the analysis, KMO = 0.823, and all KMO values for individual items were above the acceptable limit of 0.6. Barlett’s test of sphericity, χ^2^(120) = 2426.29, *p* < 0.001, indicated that correlations between measures were sufficiently large for PCA. Four components had eigenvalues larger than Kaiser’s criterion of 1 and in combination explained 71.60% of the variance (Field, 2009). Table 1 shows the factor loadings after rotation. Apart from slight deviations with respect to RNG(2) and Phi-gram measures, this factorial structure largely resembles the structure based on the paced RNG data reported by Maes et al. (2011) shown in Table 2.

**Table 1:** **Summary of principal component analysis of the data from the non-paced RNG task after orthogonal rotation (*N* = 242)**.

	Updating	Inhibition of prepotent responses	Output inhibition	Undefined
Redundancy	0.782			0.432
RNG2	0.713	0.478		
RG median	−0.674			−0.486
RG mean	−0.652		−0.461	
Coupon	0.630			0.515
Adjacency		0.885		
TPI		−0.828		
Runs		0.791		
RNG	0.593	0.645		
Phi 2			0.879	
Phi 3			0.719	0.455
Phi 4			0.570	0.556
Phi 6				0.803
Phi 5				0.637
Phi 7				0.634
RG mode				−0.546
Eigenvalues	3.200	2.817	2.392	3.047
% of variance	19.998	17.607	14.949	19.045

*Output is sorted by size and a cut-off value of 0.4 was used*.

**Table 2:** **Summary of principal component analysis of the paced RNG data from Maes et al. (2011) after orthogonal rotation (*N* = 118)**.

	Updating	Inhibition of prepotent responses	Output inhibition	Undefined
Redundancy	0.792			
RNG2		0.859		
RG median	−0.785			
RG mean	−0.586			
Coupon	0.830			
Adjacency		0.874		
TPI		−0.844		
Runs		0.478		0.769
RNG		0.874		
Phi 2			0.876	
Phi 3			0.811	
Phi 4			0.691	
Phi 6	0.423	−0.569		
Phi 5	0.445		0.462	0.521
Phi 7	0.631			
RG mode	−0.475			
Eigenvalues	3.409	3.844	2.729	1.201
% of variance	21.304	24.026	17.059	7.508

*Output is sorted by size and a cut-off value of 0.4 was used*.

Next, PCA was conducted to explore the factorial structure of all 16 randomization measures in combination with seven RQA measures, with four oblique rotated components (promax). The Kaiser–Meyer–Olkin measure verified the sampling adequacy for the analysis, KMO = 0.841, and all KMO values for individual items were above the acceptable limit of 0.7. Barlett’s test of sphericity, χ^2^(253) = 6116.29, *p* < 0.001, indicated that correlations between measures were sufficiently large for PCA. The initial extraction, before rotation, of four components explained 70.39% of the variance. Table 3 shows the factor loadings after rotation, and Table 4 shows the structure matrix. The structure matrix confirms the assumed inter-correlation between components, mainly the components updating and output inhibition.

**Table 3:** **Summary of principal component analysis of the non-paced RNG data after oblique rotation (*N* = 242): pattern matrix**.

	Updating	Inhibition of prepotent responses	Output inhibition	Undefined
Averaged diagonal		0.949		
Entropy		0.905		
Longest diagonal		0.845		
RNG		0.741		
Determinism		0.729		
RNG2	0.405	0.691		
TPI		−0.497	−4.21	−0.473
Redundancy	1.010			
Recurrence rate	0.995			
Coupon	0.777			
RG median	−0.759			
RG mean	−0.558			
RG mode	−0.495			
Phi 5	0.478			
Phi 6	0.473			
Phi 7				
Phi 2			0.892	
Phi 3			0.802	
Laminarity			0.801	
Trapping time			0.688	
Phi 4			0.608	
Runs				0.813
Adjacency	0.476			0.757
Eigenvalues	5.124	7.965	1.802	1.299
% of variance^a^	22.280	34.630	7.833	5.647

*Output is sorted by size and a cut-off value of 0.4 was used*.

*^a^% of variance before rotation*.

**Table 4:** **Summary of principal component analysis of the non-paced RNG data after oblique rotation (*N* = 242): structure matrix**.

	Updating	Inhibition of prepotent responses	Output inhibition	Undefined
Entropy		0.915		0.424
Averaged diagonal		0.913		
RNG	0.488	0.874		0.455
Determinism	0.408	0.809		
RNG2	0.503	0.797		
Longest diagonal		0.774		
TPI		−0.678		−0.620
Redundancy	0.931			
Recurrence rate	0.921			
RG median	−0.842		−0.548	
Coupon	0.826		0.485	
RG mean	−0.714		−0.572	
Phi 5	0.648		0.604	
RG mode	−0.629		−0.537	
Phi 6	0.555		0.517	
Phi 7	0.476		0.411	
Laminarity	0.560		0.893	
Phi 2			0.882	
Phi 3			0.777	
Phi 4	0.453		0.703	
Trapping time			0.646	
Adjacency		0.539		0.853
Runs				0.852

*Output is sorted by size and a cut-off value of 0.4 was used*.

Finally, PCA was conducted to explore the factorial structure of using only the recurrence quantification measures with orthogonal rotation (varimax). For the current sample, the Kaiser–Meyer–Olkin measure verified the sampling adequacy for the analysis, KMO = 0.645, and all KMO values for individual items were above the acceptable limit of 0.5. Barlett’s test of sphericity, χ^2^(21) = 1131.12, *p* < 0.001, indicated that correlations between measures were sufficiently large for PCA. Two components had eigenvalues above Kaiser’s criterion of 1 and in combination explained 71.91% of the variance. For the sample from the study by Maes et al. (2011), the KMO measure = 0.734, and all KMO values for individual items were above the acceptable limit of 0.5. Barlett’s test of sphericity, χ^2^(21) = 589.23, *p* < 0.001, indicated that correlations between measures were sufficiently large for PCA. Two components had eigenvalues above Kaiser’s criterion of 1 and in combination explained 76.34% of the variance. Tables 5 and 6 show the corresponding factor loadings after rotation.

**Table 5:** **Summary of principal component analysis of the non-paced RNG data after orthogonal rotation (*N* = 242)**.

	Inhibition of prepotent responses	Updating
Averaged diagonal	0.957	
Entropy	0.937	
Longest diagonal	0.852	
Determinism	0.730	
Laminarity		0.861
Trapping time		0.765
Recurrence rate		0.712
Eigenvalues	3.086	1.948
% of variance	44.085	27.830

*Output is sorted by size and a cut-off value of 0.4 was used*.

**Table 6:** **Summary of principal component analysis of the paced RNG data from Maes et al. (2011) after orthogonal rotation (*N* = 118)**.

	Inhibition of prepotent responses	Updating
Averaged diagonal	0.963	
Longest diagonal	0.922	
Determinism	0.917	
Entropy	0.839	
Laminarity		0.918
Trapping time		0.878
Recurrence rate		0.486
Eigenvalues	3.487	1.857
% of variance	49.818	26.523

*Output is sorted by size and a cut-off value of 0.4 was used*.

## Discussion

In the current study, we explored RNG in healthy participants by using non-linear methods to quantify performance, specifically RQA. To this aim, the performance of students on a non-paced RNG task was compared to that in an earlier sample (Maes et al., 2011), using a paced RNG paradigm. Performance was analyzed using both traditional and RQA measures. We found that the RQA measures align well within the same factorial structure that is often reported in the literature using the traditional measures. Moreover, the RQA measures explain about the same amount of variance within RNG performance, but have a more sparse interpretation than that based on the traditional measures.

Only small differences between the factorial structure of the data from the current non-paced and earlier paced RNG paradigms were found. Moreover, the interpretation of the four components of the current non-paced RNG data did not differ from the often reported factorial structure with the three-defined components (inhibition of prepotent responses, working memory updating, and output inhibition) and one undefined component. Adding RQA measures to the factorial structure did not change this interpretation and the same structure could still be subtracted. Thus, the RQA measures convey more or less the same information as the traditional measures and most, if not all, theoretical implications should be applicable to the RQA measures.

Although no significant difference in the amount of explained variance was found between the RQA and the traditional measures, the amount of extracted components did differ. Whereas the traditional measures are interpreted within the aforementioned four-component structure, the RQA measures are aligned on just two components that could be interpreted as reflecting inhibition of prepotent responses and working memory updating. Diagonal line structures are indicative of deterministic or (complex) repetitive behavior, which is closely related to inhibition. This is true for simple repetitive structures like is measured by RNG and adjacency and for complex repetitive structures, which has no equivalent conventional measure. Determinism, diagonal line length, and entropy quantifies both simple and complex repetitive structures and, therefore, inhibiting behavior. Updating of working memory content, on the other hand, is theorized to reflect equality of response usage (Towse and Neil, 1998) or keeping track of recent responses (Miyake et al., 2000). This keeping track of recent responses is quantified by redundancy, coupon, and some of the longer Phi-gram measures. In contrast, recurrence rate is the amount of all recurring numbers regardless of distance between any two numbers and this conveys, among other things, the same information as the redundancy measure and all possible Phi-gram measures without breaking the time series down into digrams. Moreover, Tables 3, 5, and 6 do confirm inhibition of prepotent responses as being one factor and, to a lesser extent, working memory updating as the other.

The benefits of this two-component interpretation over the traditional component solution are twofold. First of all, the fourth component in the component structure of the traditional measures does explain a substantial amount of variance without, however, having a satisfying interpretation. Mayhap, this variance might be attributed to the sequencing of digits on longer timescales, seen in the longer Phi-gram measures and runs. Theoretically, these measures should coincide with either working memory updating (Phi gram) or inhibition of prepotent responses (runs). However, by using summary statistics, these randomization measures capture only a derivative of order and, therefore, create an artificial fourth factor. Secondly, this two-factor solution preempts the high correlation between updating of working memory content and output inhibition (Table 4). Furthermore, Baddeley et al. (1998) theorized that output inhibition reflects an automatic process of short-term negative priming and, hence, should not be attributed to the central executive or working memory. In other words, output inhibition is an automatic process and not part of human executive behavior and should, therefore, not be interpreted as a variable in theories on executive functioning. This effect of negative priming should primarily be seen on short timescales as captured by Phi 2-gram and Phi 3-gram, whereas the contribution of working memory gets larger with increasingly longer time intervals. The current findings show this by the overall high correlation between working memory updating and output inhibition, but this correlation is non-existent for the Phi 2-gram and Phi 3-gram measures (Table 4). Laminarity and trapping time are closely related to Phi 2-gram and, therefore, this effect of negative priming limits the information gained from these RQA measures (often the percentage of recurring points forming vertical line structures is zero), which makes their loadings on the updating component hard to interpret. However, taking all this argumentation into account, the RNG task measures only two domains of executive functioning, namely inhibition of stereotyped behavior and monitoring working memory content. As mentioned earlier, the advantage of RQA over the conventional measures is that the time series is kept intact. Most conventional measures use either the frequency of digrams or a summary statistic to model RNG and, thereby, lose all information of longer timescales. In contrast, all RQA measures are based on the longest timescale available within the data. In other words, whereas conventional measures quantify only snapshots of executive behavior, RQA uses all available information and quantifies executive behavior in a more holistic manner.

To summarize, we found that the RQA and traditional randomization measures convey the same information, but the RQA measures do so in a more parsimonious way with a better interpretation. Based on these findings, we believe that RQA is a useful, if not better, alternative to the study of executive functioning using RNG. Moreover, RQA and other non-linear methods offer many more perspectives to broaden our understanding of executive functioning that is reflected in RNG performance. One of these perspectives is to include non-paced response times into our analysis, as variability of response time intervals is another source of information that tells us much about the underlying system’s properties (Holden et al., 2009; Wijnants et al., 2009). In closing, it is important to keep the temporal structure of behavior intact, and for future reference, we will abide to this claim by embedding our discussion on RNG into a framework of interaction-dominance and complexity science.

## Conflict of Interest Statement

The authors declare that there is no conflict of interest in the writing and publication of this article.
